# Hydroxyapatite Deposition Disease as Cause of Atraumatic Shoulder Pain: A Case Report

**DOI:** 10.5811/cpcem.35244

**Published:** 2025-01-12

**Authors:** Hong Diem Truong, Andrew Gonedes, Jason Haidar, Kevin Wilson, Michael Remaly, Eric Boccio

**Affiliations:** * Memorial Healthcare System, Department of Emergency Medicine, Hollywood, Florida; † Florida International University, Herbert Wertheim College of Medicine, Miami, Florida

**Keywords:** Hydroxyapatite deposition disease, HADD, Milwaukee shoulder, shoulder pain, musculoskeletal pain

## Abstract

**Introduction:**

Hydroxyapatite deposition disease (HADD) is caused by the presence of hydroxyapatite crystals in periarticular spaces oftentimes leading to inflammation, pain, and decreased range of motion.

**Case Report:**

A 40-year-old right hand dominant female presented with three days of atraumatic right shoulder pain. Radiographs of the right shoulder were negative. Computed tomography revealed a hydroxyapatite deposit adjacent to the acromioclavicular joint. The patient was managed with nonsteroidal anti-inflammatory drugs and a referral to orthopedic surgery.

**Conclusion:**

Many instances of HADD will not be diagnosed on plain radiographs, and heightened awareness will provide confidence when ordering confirmatory imaging. Management is typically conservative, however, referral to orthopedic surgery is recommended to ensure improvement and to assess the need for more invasive procedures.

## INTRODUCTION

Hydroxyapatite deposition disease (HADD) is a systemic condition characterized by the deposition of hydroxyapatite crystals in periarticular tissues, such as tendons, bursae, and joint capsules, leading to localized pain, swelling, and restricted joint movement. Hydroxyapatite deposition syndrome commonly presents in the shoulder, typically involves the supraspinatus tendon, and can often be confused with other shoulder pathologies, such as gout or pseudogout, arthritis, malignancy, or rotator cuff injury. Diagnostic testing for HADD typically begins with plain radiography, which can reveal calcifications of varying sizes and shapes in affected areas. In more complex cases or to better assess soft tissue involvement, advanced imaging techniques such as computed tomography (CT) or magnetic resonance imaging (MRI) may be employed. These imaging modalities are particularly useful in identifying specific characteristics of calcifications and differentiating HADD from other conditions. Therapeutic management of HADD varies depending on the severity and symptoms. Initial treatment often includes analgesics, cryotherapy, physical therapy, and steroid injections. In more severe cases, or when conservative management fails, surgical intervention may be necessary to remove the deposits. The disease can progress through several stages, each with varying degrees of inflammation and severity of pain. Treatment is tailored based on the stage and progression of disease as well as the severity of associated symptoms. We present a case of HADD as a cause of atraumatic shoulder pain presenting in the emergency department (ED) and discuss the etiology, clinical presentation, diagnostic workup, and standard therapeutic management options including both conservative and interventional approaches.

## CASE REPORT

A 40-year-old right hand dominant female with a past medical history of anemia, anxiety, depression, uterine fibroids, idiopathic intracranial hypertension, migraine headaches, and supraventricular tachycardia status post cardiac ablation and past surgical history of breast reduction surgery, four Cesarean sections, robotic laparoscopic hysterectomy, bilateral salpingectomy, bilateral tubal ligation, and right ovarian cystectomy presented to the ED with right shoulder pain. The patient stated that the shoulder pain began three days earlier and was present upon awakening from sleep. The patient denied trauma, overuse, fall, and seizure. The patient reported reduced range of motion with inability to abduct, externally rotate, and flex the shoulder due to pain. The patient denied any history of shoulder surgery, previous injury, and past dislocation. The patient denied neck pain and numbness, paresthesia, and weakness of the right upper extremity.

The patient’s vital signs at triage were within normal limits for age (blood pressure, 126/83 millimeters (mm) of mercury; pulse, 83 beats per minute; respiratory rate, 18 breaths per minute; and temperature, 36.3 °Celsius, oral). Physical examination was remarkable for a deformity of the right shoulder with swelling and tenderness over the acromioclavicular joint and proximal third of the humerus. There was no evidence of hyperemia or erythema. The patient experienced pain with active and passive range of motion of the right shoulder. The radial pulse was strong and regular when palpated, and capillary refill was less than three seconds. Two-point discrimination in the right fingertips was intact, and there was no evidence of decreased grip strength. The neck was supple and exhibited full range of motion; there was no midline cervical tenderness, and Spurling test was negative. There was no tenderness with palpation of the midshaft and distal humerus, radius, and ulna. The elbow and wrist joints exhibited full range of motion without resistance or pain. There were no gross signs of trauma on visual inspection of the right upper extremity.

**Image 1:**
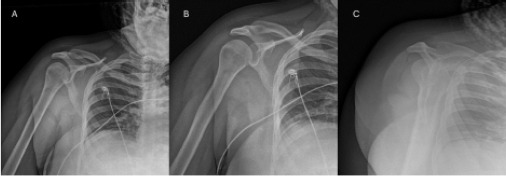
Three-view radiographs of the right shoulder demonstrating no fracture or dislocation in the A) anteroposterior externally rotated, B) anteroposterior internally rotated, and C) scapular Y projections.

The patient was given morphine 4 milligrams (mg) intravenous (IV) twice for a total of 8 mg and ketorolac 15 mg IV. The differential diagnosis included rotator cuff tendonitis and complete or partial tear, bursitis, osteoarthritis, adhesive capsulitis, and labrum tear. Three-view radiographs of the right shoulder showed no fracture or dislocation (Image 1).

**Image 2:**
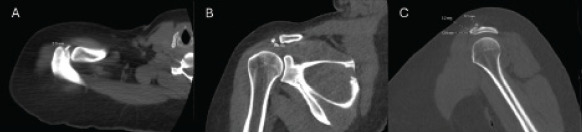
Computed tomography imaging without intravenous contrast of the right shoulder demonstrating A) axial, B) coronal, and C) sagittal series. A 13×3×5 millimeter calcific deposit overlying the anterior aspect of the acromioclavicular joint adjacent to the acromion consistent with hydroxyapatite deposition disease (dotted lines) is seen. No fracture or dislocation was visualized.

Given the patient’s physical examination findings, unremarkable radiography, and continued complaints of pain and inability to range the shoulder despite analgesics, contiguous 1.25 mm thick transaxial CT imaging of the right shoulder without prior administration of IV contrast material was obtained. Computer generated coronal and sagittal reformations were performed. A 13×3×5 mm calcific deposit was identified along the anterior aspect of the acromioclavicular joint adjacent to the acromion suggestive of HADD. The acromioclavicular joint was intact and overlying subcutaneous edema and heightened fat stranding was visualized without a discrete fluid collection. No fracture or dislocation was identified (Image 2).

**CPC-EM Capsule**

What do we already know about this clinical entity?

*Hydroxyapatite deposition syndrome (HADD) involves musculoskeletal pain due to inflammation caused by crystals in tendons, bursae, and joint capsules*.

What makes this presentation of disease reportable?

*Atraumatic shoulder pain and swelling is a common reason for presentation to the emergency department and has a broad differential*.

What is the major learning point?

*Hydroxyapatite deposition syndrome is a relatively uncommon cause of atraumatic shoulder pain that may not be distinguishable on plain radiographs*.

How might this improve emergency medicine practice?

*Diagnosis of HADD may require ordering of computed tomography and is treated with anti-inflammatories, physical therapy, extracorporeal shock wave therapy, and barbotage*.

A shoulder sling was applied, and the patient was discharged with an ambulatory referral to orthopedic surgery and prescriptions for naproxen, orphenadrine, and hydrocodone-acetaminophen.

## DISCUSSION

The etiology of HADD involves the deposition of hydroxyapatite crystals in tendons, bursae, and joint capsules, leading to inflammation that manifests as pain, swelling, and reduced mobility. Hydroxyapatite deposition syndrome is estimated to affect 2.7% of adults, is more common in females, and has a peak incidence in the fourth to sixth decades of life.^1^ The shoulder is the most commonly affected site, and within the shoulder, the supraspinatus is often involved due to its anatomical position and susceptibility to microtrauma and ischemia; periarticular tissues, especially tendons, may also be involved.^2^ The hip, elbow, wrist, and knee are the subsequent most common sites, and HADD rarely involves the ankle, foot, and fingers.^1^

Hydroxyapatite deposition disease develops over four stages: the pre-calcific stage marked by fibrocartilaginous transformation due to vascular or mechanical injury, the formative stage during which the transformed tissue is replaced with calcium deposition, the resting stage where additional fibrocartilaginous tissue borders the calcifications, and finally, the resorptive stage which involves the extravasation of the calcifications into adjacent tissues. Hydroxyapatite deposition disease is frequently an incidental finding in its early stages as it is often asymptomatic. As HADD progresses, pain develops, and, therefore, HADD typically presents to the ED and is diagnosed in its advanced stages.^3^

Typically, patients suffering from HADD present clinically with atraumatic monoarticular pain, although some patients may have a history of prior injury to the joint. Swelling and erythema may be present. If the crystal size becomes large enough or is positioned within the joint space, articulation will be painful and range of motion will be limited. Over time, HADD may result in joint destruction.^4^ The diagnosis is often established via high clinical suspicion and confirmatory diagnostic imaging. Radiographs are highly sensitive but can miss subtle amorphous calcifications, especially when there are overlapping structures. Ultrasonography will reveal echogenic foci with posterior acoustic shadowing but utility may be limited in certain locations due to technical difficulties associated with manipulating and positioning the probe. Computed tomography and MRI are highly sensitive for detecting calcification and pericalcific inflammation and, hence, they are most useful in confirming the diagnosis of HADD.^5^ However, these imaging modalities are infrequently used in acute care settings due to the nonemergent nature of the presentation.^6^

Therapeutic management strategies vary based on disease stage and severity of symptoms. A conservative approach involving nonsteroidal anti-inflammatory drugs (NSAIDs), cryotherapy, rest, and physical therapy has been shown to provide clinically significant improvement in 72% of patients with calcific tendinitis of the shoulder.^7^ Lack of resorption of the hydroxyapatite deposits or penetration of liquified crystals into surrounding soft tissue may result in a local inflammatory reaction that is amenable to needling and corticosteroid injections.^8^ For refractory cases lasting longer than six months or those involving functional impairment, barbotage, ultrasound-guided percutaneous needle aspiration and lavage, and extracorporeal shock wave therapy have been shown to reduce the size of the calcium deposits.^9^ Ultrasound-guided barbotage which involves the needling down of crystal deposits and lavage to remove the resultant fragments has been shown to effectively remove calcium deposits in up to 91% of patients.^10^ Lastly, surgical excision should be considered if minimally invasive procedures prove ineffective.^7^ A cost-effectiveness analysis demonstrated that ultrasound-guided barbotage was the most cost-effective strategy when compared to low-energy extracorporeal shock wave therapy and surgery, and conservative management was considered reasonable in appropriate clinical settings.^11^

This case describes an atypical presentation of HADD as radiographs failed to capture the hydroxyapatite crystal deposition and CT imaging was required. The calcific deposits were found to be adjacent to the acromion just anterior to the acromioclavicular joint. While the shoulder was the only joint affected, the supraspinatus was spared. Our patient was female and in her fifth decade of life, consistent with common demographic risk factors for HADD. The patient presented with atraumatic monoarticular joint pain and an inability to range the shoulder due to pain likely secondary to the size, location, and associated inflammation and swelling caused by the relatively large 13×3×5 mm hydroxyapatite deposit. Our patient was managed with analgesics and NSAIDs and provided with an ambulatory referral to orthopedic surgery. Per review of the patient’s medical record, there were no future minimally invasive procedures or surgery scheduled to date.

## CONCLUSION

This case report emphasizes the importance of distinguishing HADD from other causes of shoulder pain and highlights the role of imaging in establishing a diagnosis and guiding management. While the most severe stages of HADD may be detected on radiographs, visualization of hydroxyapatite crystals within or adjacent to periarticular structures typically requires CT or MRI. While conservative management including NSAIDs and physical therapy is appropriate in most clinical scenarios, minimally invasive procedures such as needling, barbotage, and extracorporeal shock wave therapy may be necessary for moderate and severe cases or in refractory cases. Surgical intervention is considered for the most severe cases that result in significant disability or when other methods fail.
